# Phage-Based Control of *Listeria innocua* in the Food Industry: A Strategy for Preventing *Listeria monocytogenes* Persistence in Biofilms

**DOI:** 10.3390/v17040482

**Published:** 2025-03-27

**Authors:** Anna Zawiasa, Marcin Schmidt, Agnieszka Olejnik-Schmidt

**Affiliations:** Department of Food Biotechnology and Microbiology, Poznan University of Life Sciences, Wojska Polskiego 48, 60-627 Poznan, Poland; anna.zawiasa@up.poznan.pl

**Keywords:** bacteriophages, biofilms, *Listeria innocua*, *Listeria monocytogenes*, safety

## Abstract

*Listeria innocua*, though considered non-pathogenic, frequently coexists with *Listeria monocytogenes* in industrial environments, aiding its survival in biofilms. These biofilms pose a significant challenge in food processing facilities, as they protect bacteria from disinfectants and facilitate their spread. The aim of this review was to identify bacteriophages as a promising method for eliminating *Listeria* biofilms from the food industry. Lytic bacteriophages show great potential in combating *Listeria* biofilms. Commercially available products, such as PhageGuard Listex™ (P100) (Micreos Food Safety, Wageningen, The Netherlands), effectively reduce both *L. monocytogenes* and *L. innocua* in food products and on production surfaces. Additionally, phage-derived enzymes, such as endolysins, can degrade biofilms, eliminating bacteria without compromising food quality. The following article highlights that although bacteriophages present a promising biocontrol method, further research is necessary to assess their long-term effectiveness, particularly regarding bacterial resistance. To maximize efficacy, a combination of strategies such as phage cocktails and disinfectants is recommended to enhance biofilm eradication and minimize food contamination risks.

## 1. Introduction

*Listeria* species are a widely distributed group of Gram-positive, facultative anaerobic bacteria. As of 2021, 27 species of *Listeria* have been documented [1], with *L. monocytogenes* and *L. innocua* as the most prevalent species [2]. Isolates of both species are the most commonly identified in food processing plants, especially posing a problem in animal-based industrial facilities [3]. *L. monocytogenes* is a well-recognized pathogen harmful to both animals and humans [1,2,3]. It leads to listeriosis, a disease that particularly affects pregnant women, newborns, the elderly, and those with compromised immune systems [1,4,5]. This infection can result in septicemia, endocarditis, meningitis [5], with a reported mortality rate of 20–30% [6,7]. Due to the severity of the disease, *L. monocytogenes* is considered one of the most significant foodborne pathogens in terms of both economic impact and public health concerns [8]. The incidence of listeriosis is mainly linked to consuming contaminated foods, like nonpasteurized milk, soft cheeses, vegetables, raw and processed meats, seafood, and ready-to-eat (RTE) products [2,8,9]. *L. monocytogenes* is remarkably resilient to challenging environments, including osmotic and cold stress, low pH levels, desiccation, and competition from other microorganisms [2].

In contrast, *L. innocua* is non-pathogenic and non-hemolytic [3,10]. *L. innocua* shares an evolutionary connection with *L. monocytogenes*. It is suggested that *L. innocua* and *L. monocytogenes* have evolved from a common virulent ancestor [3,11], with their primary differences arising from the loss of virulence genes in *L. innocua*. This theory is supported by the presence of atypical *L. innocua* isolates that retain virulence factors [11] including *Listeria* pathogenic island 1 (LIPI-1), LIPI-3, and LIPI-4 [3,9,12]. The presence of *hly* gene, being part of LIPI-1 in the *L. innocua* genome, is responsible for the hemolytic phenotype observed in atypical isolates [13,14]. Atypical strains could potentially act as reservoirs of virulence genes, which may be transferred to other species within the genus by horizontal gene transfer [13,15]. Some atypical hemolytic *L. innocua* strains have been isolated from fish and seafood in Asia as the first described atypical *L. innocua* strain [16], pork and swine slaughterhouses in North America [13], and poultry in Europe [17]. The atypical hemolytic *L. innocua* can cross the intestinal barrier and spread to internal organs [9], but its virulent potential is limited when compared to the virulent strains of *L. monocytogenes* [14]. There have been documented cases linking atypical *L. innocua* strains to severe conditions in immunosuppressed individuals [14,18], such as fatal bacteremia [19], meningitis [20], neonatal sepsis [21], meningoencephalitis in a three-year-old boy [22] and joint infection in a case of total knee replacement [23] as well as two cases of nervous listeriosis in ruminants [10]. These cases suggest that *L. innocua* may possess pathogenic potential under specific conditions [1,12] and will challenge risk management in the food chain [1,2]. The purpose of this review was to examine the phenomenon of coexistence among different *Listeria* species, particularly *L. monocytogenes* and *L. innocua*, in biofilms found in the food industry. Additionally, the review sought to emphasize the potential of bacteriophages as an innovative strategy for eradicating *Listeria* biofilms.

## 2. The Occurrence of *L. innocua* in the Food Industry

*L. innocua* is a ubiquitous bacterium commonly found in a wide range of natural and industrial environments, including food and food processing facilities [1,10,14] like pork slaughterhouses and a meat market [13]. *L. innocua* has also been detected in milk and dairy products [2,18]. *L. innocua* coexists in the same food products and environmental settings as *L. monocytogenes* [3,24,25]. These two species are often detected together in the same samples [2,26], with *L. innocua* being more common in food processing environments than *L. monocytogenes* [2,3,15] and exhibiting faster growth than *L. monocytogenes* in food and other media, masking the presence of *L. monocytogenes* and leading to potentially false-negative results [15,27]. Due to its ecological cohabitation, genomic, and phenotypic similarities, *L. innocua* is regarded as an effective indicator organism [1,2,28,29]. Its detection in food products, as well as food contact and noncontact surfaces, suggests that conditions might be favorable for the growth or survival of more pathogenic *L. monocytogenes* [9]. Therefore, it is crucial to understand how *L. innocua*, in addition to *L. monocytogenes*, reacts to interventions aimed at controlling *L. monocytogenes* in food processing plants [25].

## 3. Disinfection in the Food Industry

Ensuring effective sanitation in food processing environments is crucial for reducing product contamination [30,31]. Due to the ability of *Listeria* spp. to persist for extended periods on equipment and within food industry settings, cross-contamination from production environments to food during processing is regarded as the primary route of contamination for this pathogen [32]. Currently, surfaces prone to biofilm contamination are disinfected using conventional agents like sodium hypochlorite and quaternary ammonium compounds (QACs), such as benzalkonium chloride [30,33]. In these conditions, bacteria are continuously exposed to specific concentrations of disinfectants, potentially leading to selection pressure that prompts initially susceptible bacteria to adapt and develop resistance [30,34,35]. Several studies have shown that *Listeria* spp. isolated from food products and food environments exhibit a higher incidence of QACs tolerance compared to those isolated from humans, or other environmental samples [33,36,37,38]. Disinfectant resistance plays a crucial role in bacterial survival within contaminated environments [34], and it can increase the risk of persistence of *L. monocytogenes* and other *Listeria* spp. in food processing facilities [7,8,39]. One of the most recognized mechanisms for developing resistance is biofilm formation [8,40,41]. *Listeria* spp. within biofilms exhibit lower sensitivity to biocides compared to their planktonic forms [40,42]. While many antimicrobials used in the food industry can reduce and inactivate *Listeria* spp., risks still exist related to the detachment and regrowth of the cells. Consequently, biofilms continue to be a significant concern [7,40,43].

## 4. Biofilms

Bacterial biofilms are organized communities of cells encased in self-generated extracellular polymeric substances (EPSs), which adhere to both biotic and abiotic surfaces, like food surfaces as well as in processing environments and equipment [43,44,45]. In most bacterial biofilms, microorganisms constitute only 10% of the dry weight, while the proportion of EPSs exceeds 90% [46]. EPSs are composed of a diverse array of extracellular polysaccharides, proteins, lipids, and nucleic acids, including extracellular DNA (eDNA) [47,48]. This biofilm structure facilitates genetic material exchange, nutrient supply, and protection from stressors such as disinfectants and other antimicrobial agents [43,46].

The formation of bacterial biofilm is a multi-step and dynamic process that includes initial reversible attachment [49], followed by irreversible attachment [50], microcolony formation, maturation, and finally dispersal [30]. Dispersion is not just the final stage of biofilm development; it also marks the start of a new biofilm life cycle [47]. Effective coordination among various bacteria within the biofilm is crucial and depends on chemical communication between cells [47,48]. Quorum sensing, a mechanism that facilitates cell-to-cell communication, synchronizes gene expression based on population density. This process allows bacteria to gauge their population density by accumulating specific signaling molecules, thereby enabling them to adjust their survival strategies through differential gene expression [51].

The attachment of *Listeria* spp. and their biofilm formations can be influenced by various factors like growth stage of the bacteria, the characteristics of the specific strain, temperature, the chemical and physical properties of the attachment substrate, and the presence of other microorganisms [45,46]. Pathogenic bacteria such as *L*. *monocytogenes*, which is often linked to foodborne illnesses, form biofilms on surfaces within the food supply chain, posing a significant public health concern [46]. The formation of biofilms by *Listeria* spp. on surfaces that come into contact with food is recognized as a significant route for the persistence of this pathogen and the resulting contamination of products [42,43].

## 5. Coexistence of *L. innocua* and *L. monocytogenes* in Biofilms

In biofilms, the coexistence of multiple microbial species is observed [8,48,52]. The ability to form biofilms has already been researched. When grown in pure culture, *L. monocytogenes* adhered to surfaces more effectively than *L. innocua*. However, in mixed biofilms, *L. innocua* outgrew *L. monocytogenes*. The hydrophobic properties of the cell surface of *L. innocua* were found to be more electronegative than that of *L. monocytogenes*, which may have led to increased initial attachment for *L. innocua* and demonstrated stronger competitive growth compared to *L. monocytogenes*. These findings suggest that *L. innocua* influenced the attachment of *L. monocytogenes* [26].

Generally, *L. monocytogenes* is not an efficient biofilm former, which has led to the proposal that these strains might rely on a primary colonizing bacterium like *L. innocua* to establish a biofilm consortium on surfaces [53]. *L. innocua*, like *L. monocytogenes* can attach to and develop biofilms on various materials used in food processing equipment, including polymers, plastic, glass, polystyrene, Teflon, rubber, and stainless steel [44,53,54]. A study comparing the biofilm formation capabilities of various *Listeria* spp. revealed that *L. innocua* is capable of forming strong biofilms across a range of temperatures [55]. Di Bonaventura’s 2008 study revealed that both *L. monocytogenes* and *L. innocua* exhibited strong adhesion at 37 °C and 25 °C. However, *L. innocua* showed superior performance at lower temperatures like 12 °C. This characteristic contributes to its high contamination rates and its higher incidence and prevalence than *L. monocytogenes* in food production environments [44,46,54].

It is hypothesized that *L. innocua* cohabits with *L. monocytogenes* within biofilms, thereby enhancing the pathogen’s defense against disinfectants. Removing *L. innocua* from these biofilms may potentially aid in eradicating *L. monocytogenes.* Elimination biofilms composed of *Listeria* spp. in the food processing environment is crucial, as these biofilms on food processing surfaces can lead to cross-contamination and increase the prevalence of the pathogen within the food system [44,53,54]. Improving our understanding of the interactions between various antimicrobials and biofilm cells on different surfaces is necessary. The complex nature of biofilms and their cells’ capacity to firmly adhere to inaccessible surfaces diminishes the effectiveness of currently used disinfectants. Therefore, innovative methodologies or strategies are necessary to address the issue of biofilm development [42,43]. Alternative methods have been investigated for controlling *Listeria* biofilms, including the use of enzymes such as proteinase K, pronase, cellulase, pectinase, lysozyme, phospholipase, peroxidase, and chitinase for biofilm dispersal. However, for these enzymatic approaches to be effective, they typically need to be combined with bactericidal treatments [56]. One of the promising solutions is using bacteriophages as a green biocontrol method to eradicate *L*. *monocytogenes* from biofilms [48,57].

## 6. Bacteriophages

Bacteriophages (phages) are the most abundant microorganisms on the planet. They can be found widely in nature and every environment inhabited by bacteria, such as water, soil, sewage, food, and the human gut [58]. Phages are viruses that specifically target and destroy bacteria [58,59]. Based on their replication cycles, bacteriophages can be classified into two groups: lytic and lysogenic [57]. Only phages with a lytic cycle can be utilized as biocontrol agents for microorganisms in food and food environments [60]. Lytic phages attach to bacterial cells, inject their genetic material, replicate within the host, assemble new viral particles, and ultimately lyse the bacterial cell wall by lytic enzymes leading to bacterial cell death [57].

To gain approval as a biocontrol product for food, a bacteriophage must satisfy several stringent conditions. Above all, bacteriophage, as mentioned, must undergo the lytic cycle leads to bacterial cell death and targets a wide range of strains. Bacteriophages used in this process cannot transfer virulence or toxicity genes between bacteria through transduction, nor can they encode pathogenicity genes. They must not alter the organoleptic properties of the final products, not influence microorganisms other than those targeted, and must remain stable during storage and application [61,62]. They are regarded as safe for humans due to their high specificity to hosts, ensuring they do not impact human microbiota or the starter cultures, important for food production (useful in fermented products) [60]. For instance, phage P100, targeting *Listeria* spp., was found to have no impact on the functional lactic acid bacteria in the cheese, nor did it alter the characteristics of the product [63].

Bacteriophages can target and eliminate pathogenic microorganisms like *L*. *monocytogenes* in various food products, including raw meat, fish, milk, cheeses, fruits, vegetables, and ready-to-eat foods [64,65,66,67,68,69,70], as well as they can be used to reduce or eliminate the pathogen in biofilm on surfaces within food processing facilities [67,71,72].

## 7. Bacteriophages and Biofilms

As natural enemies of bacteria, phage-based treatments can combat biofilms through multiple mechanisms. Phages can prevent biofilm formation as well as destroy bacterial hosts within biofilm. The presence of specific receptor sites on the surface of biofilm bacteria is essential for phage attachment and infection [48]. They are also capable of penetrating existing biofilms and disrupting their structure [46]. Phages can generate endolysins that effectively degrade exopolysaccharides, a major component and protective element of the biofilm matrix. This enables the phages to infiltrate and disrupt the biofilm, freeing individual bacteria and increasing their susceptibility to other antimicrobial agents [29,73,74]. The accessibility of phages to biofilm is influenced by the physiological condition, and physical environment of various microorganisms. Bacteriophages can self-replicate within host bacteria, leading to higher densities in biofilms and rapid population growth. This enables a single dose of preparation to effectively eliminate pathogens. These properties make bacteriophages advantageous as antibacterial agents for biofilm elimination [73,74].

## 8. *Listeria* spp. Bacteriophages

Two phage-based products are commercially available for targeting *L. monocytogenes* in food products and processing environments. In 2006, the first approval for a bacteriophage preparation to be used directly in the food supply was issued by the FDA for the *L. monocytogenes*-specific cocktail ListShield™ as a food additive. ListShield™ (LMP-102) is a cocktail of six lytic phages [75]. Next, the FDA approved another *Listeria*-specific preparation, PhageGuard Listex™, as a Generally Recognized as Safe (GRAS) substance [57]. PhageGuard Listex™, which comprises a single broad-host-range phage, P100 [67]. It is worth mentioning that P100 is closely related to other *Listeria* phage A511 [65]. Most experiments utilized these products, with only a few studies focusing on other bacteriophages. In numerous experiments, *L. innocua* is employed as a surrogate for *L. monocytogenes*, enabling researchers to assess the effectiveness of bacteriophages against *L. innocua* as well [17].

### 8.1. Phage-Based Biocontrol of L. innocua in Foods

There were attempts suggesting that phage P100 can be used against *L. innocua* in food products. The study by Colás-Medà et al. [60] highlighted the effectiveness of the bacteriophage P100 in eradicating *L. innocua* in three types of ready-to-eat (RTE) foods: sliced cooked ham, fresh cheese, and fuet (products linked to listeriosis). Three doses of PhageGuard Listex™ (1%, 0.5%, and 0.2%) were tested. In sliced cooked ham stored at 13 °C, the number of *L. innocua* decreased to 3.9 logarithmic units compared to 6.8 logarithmic units in the control sample when treated with PhageGuard Listex™. Under modified atmosphere packaging (MAP) at 4 °C, PhageGuard Listex™ treatments reduced *L. innocua* to undetectable levels. In fresh cheese, all evaluated doses of PhageGuard Listex™ reduced *L. innocua* populations below the detection limit. However, only the 0.5% and 1% PhageGuard Listex™ treatments prevented *L. innocua* regrowth after storage. In fuet, Listex™ P100 caused a reduction of the *L. innocua* population by around 1 logarithmic unit compared to the control at the end of shelf life. Research findings indicate that PhageGuard Listex™ exhibited effective anti-listerial activity, but its efficacy against *L. innocua* varies depending on the food matrix and the physicochemical properties of the product [60].

In a study by Lewis et al. [76] a variety of *Listeria* strains were tested for sensitivity to P100. The study found that while P100 exhibited activity against many pathogenic and non-pathogenic *Listeria* strains, its effectiveness varied. For instance, the efficiency of the plaquing of P100 against the strain *L. innocua* DPC 3372 was 0.83 logarithmic units, whereas P100 showed no efficacy against another strain, *L. innocua* FA2039. These results indicate a clear strain dependence of phage P100 [76]. This strain dependence is a critical factor in determining the overall efficacy of phage P100 in food safety applications. Researchers have also explored the use of other bacteriophages to remove *L. innocua* from food. In the study by Zhou et al. [77], phage SH3-3, isolated from sewage, was characterized and assessed for its effectiveness against *Listeria* spp. The study found that phage SH3-3 has lytic activity not only against *L. monocytogenes* but also against *L. innocua* and *L. welshimeri*, indicating its broad host range [77]. The above studies indicate that *Listeria* phages P100 and SH3-3 have a broad host range within the genus *Listeria*, demonstrating the effectiveness of the phages in eliminating both *L. monocytogenes* and *L. innocua* from food products.

On the other hand, some studies demonstrate the effectiveness of phages against *L. monocytogenes*, but not against *L. innocua*. The study by Bigot et al. [68] found that the phage, which was morphologically similar to phage A511 isolated from sheep feces, infected strains of *L. monocytogenes*, *L. ivanovii*, and *L. welshimeri*, but did not infect *L. grayi* or *L. innocua* [68]. Similarly, in the Stone et al. [74] study, the phage vB_LmoH_P61 (P61), isolated from grass silage, exhibited high serotype dependence, successfully infecting six out of nine tested *L. monocytogenes* serogroups. However, phage P61 was unable to infect *L. innocua* strains [74]. Finally, the study by Li et al. [78], highlights the effectiveness of phage LP8, sourced from slaughterhouse sewage, in eliminating *L. monocytogenes* and *L. welshimeri* but shows no effectiveness against *L. innocua* [78]. These studies suggest that while some tested phages exhibit a broad host range, effectively targeting both *L. monocytogenes* and *L. innocua* to eliminate multispecies food contamination, others have a much narrower spectrum of activity and must be carefully selected for specific bacterial strains.

### 8.2. Inhibition of L. innocua on Surfaces

Phages can be used to eliminate or reduce *L. monocytogenes* and *L. innocua* biofilms on surfaces in food processing plants. In the study by Reinhard et al. [72], various surfaces (stainless steel, thermoplastic belting, and epoxy flooring) were tested for *L. innocua* reduction using phage P100 under different conditions: temperature (4 °C and 20 °C), phage concentration (1% and 5%), and exposure time (1 h and 3 h). *Listeria* reduction was observed on all three materials under all tested conditions. Temperature did not affect *Listeria* reduction on epoxy flooring, but greater reductions were observed on stainless steel and thermoplastic belting at 20 °C compared to 4 °C. The 5% concentration led to significantly greater reductions in *Listeria* levels on stainless steel and belting materials. On epoxy flooring, reductions were similar for both phage concentrations. A longer exposure time of 3 h resulted in higher reductions, with the greatest reductions observed on stainless steel treated with 5% phage concentration for 3 h at 20 °C [72]. Phage P100 has proven to be highly effective in removing both *L. monocytogenes* and *L. innocua* from production environments.

### 8.3. Using of Endolysin Against L. innocua

As mentioned earlier, endolysins, lytic enzymes originating from bacteriophages, disintegrate bacterial cell walls during the lytic phase of phage infection [79,80]. These enzymes have emerged as promising new-generation biocides, particularly for use as disinfectants [29]. Endolysins from phages that specifically target *Listeria* spp. are considered an optimal choice [79].

The study by Romero et al. [80] presents the structural and functional analysis of the *Listeria* phage endolysin Ply40, which targets the sugar components of the *Listeria* cell wall. The C-terminal cell wall binding domain (CBDP40) of Ply40 exhibits high affinity in binding to *Listeria* cell surfaces, recognizing strains of all *Listeria* spp., including *L. monocytogenes* and *L. innocua*, and demonstrating its lytic activity against these bacteria. The results indicate that Ply40 possesses broad-range specificity across this genus [80].

The potential of using phage endolysins to eliminate *Listeria* spp. biofilms have also been suggested. PlyLM showed applicability to planktonic *L. monocytogenes* and *L. innocua* cells, effectively reducing the monolayer biofilm to the same extent as lysozyme and proteinase K. This demonstrates the efficacy of endolysins in targeting biofilms composed of various *Listeria* spp. This is also noteworthy, as PlyLM shares 90% sequence identity with a putative *L. innocua* amidase of similar size [81]. However, because the biofilms were only cultured for 24 h at 37 °C, further investigation is needed to assess the effectiveness of these enzymes under different conditions, such as lower temperatures that better mimic real-world conditions in food processing plants [81].

In the study by Talens-Perales et al. [29] the potential of using endolysin A10, an enzyme with amidase activity derived from *Listeria* phage vB_LmoS_188, for eliminating *Listeria* spp. was highlighted. Endolysin A10 has a high identity (95%) with an endolysin from phage vB_LmoS_293, which has muralytic activity and can prevent biofilm formation on abiotic surfaces in *L. monocytogenes* [82]. A10 showed antibacterial activity against *L. innocua*, reducing viable cells by 1.2 log whereas *L. monocytogenes* exhibited higher resistance to A10, with a reduction of 0.7 log. Although most cell wall-related genes in *L. monocytogenes* have corresponding orthologs in *L. innocua*, some genes are unique to each strain, which may explain differences in response. A10′s ability to target various *Listeria* spp., despite differing sensitivities, suggests it has broad substrate specificity within the *Listeria* genus [29].

In a study by Zhang et al. [79] the antimicrobial activity of LysZ5, a *Listeria* phage endolysin from phage FWLLm3, was evaluated against *Listeria* spp. in soy milk at refrigeration temperatures. The DNA sequence of LysZ5 showed substantial similarity (96% identity) to the endolysin Ply511 from *Listeria* phage A511 [79,83]. LysZ5 caused lysis in *L. monocytogenes, L. innocua,* and *L. welshimeri,* indicating a broad lytic spectrum against *Listeria* spp. [79]. The above studies demonstrate the significant effectiveness of *Listeria* phage endolysins, including Ply40, PlyLM, A10, and LysZ5, in eliminating both *L. monocytogenes* and *L. innocua* from food products as well as surface biofilms in food production facilities.

Due to their proteinaceous nature and highly specific enzymatic activity targeting the chemical bonds that maintain the integrity of bacterial cell walls, they do not compromise the safety or sensory qualities of food products. However, the degradation of the bacterial cell wall by phage endolysins does not always lead to cell death. *Listeria* can survive the action of endolysins by transitioning to a cell wall-deficient state known as the L-form. Consequently, while endolysins can be effective in limiting *Listeria* proliferation in food environments, they may not be sufficient for the complete eradication of the pathogen [29,84].

## 9. *Listeria* Resistance to Phages

Despite these promising results, further research is necessary to assess the long-term effectiveness of phage, particularly regarding the development of phage resistance. There is concern that the extensive use of these bacteriophage treatments might eventually result in the emergence and selection of phage-resistant mutants [85]. Bacteria can evolve mechanisms to avoid phage attacks, such as CRISPR-Cas systems, alterations and mutations of surface receptors, or the production of substances that block phage attachment [86]. This phenomenon necessitates continuous monitoring. To minimize the likelihood of phage resistance development, several measures can be taken. First, phage-treated products should not re-enter the production cycle. This can be achieved by administering phages to products just before packaging, thereby preventing contamination and the emergence of phage-resistant bacteria within the production environment. Additionally, using phage cocktails with a wide range of activities can hinder resistance development [87]. The EFSA emphasized that this bacteriophage should serve solely as a supplementary measure to ensure microbial food safety, it must be combined with the application of good hygienic practices and good manufacturing practices and should not replace these essential protocols [88]. Given the potential for phage resistance, the most effective results are achieved by combining phage therapy with other strategies. These include the use of endolysins, disinfectants, and robust hygiene practices. An integrated approach can enhance the efficacy of biofilm eradication and reduce the risk of food contamination by pathogens.

## 10. Summary and Future Perspectives

*L. innocua* is prevalent in both natural and industrial settings, particularly within food processing facilities. Even though it is considered non-pathogenic, it often coexists with *L. monocytogenes* within biofilms, which can enhance its survival and complicate the eradication of this pathogen. Research indicates that *L. innocua* is better adapted to environmental conditions, demonstrates a greater ability to form biofilms at low temperatures, and may serve as a reservoir for resistance genes. It is hypothesized that *L. innocua* determines the ability of *L. monocytogenes* to survive in the biofilm actively metabolizing disinfectants and making the environment below suitable for pathogen persistence. Examples of multispecies cooperation to promote resistance to antimicrobial agents [89,90] or withstand environmental factors [91] have already been described. Consequently, the elimination of biofilms containing *L. innocua* may significantly improve the control of *L. monocytogenes.* Bacteriophages, especially lytic ones, are highly effective in eliminating both *L. innocua* and *L. monocytogenes* from surfaces in the food industry. Commercial products such as PhageGuard Listex™ (P100) have shown the ability to reduce biofilms on various surfaces (stainless steel, plastics). In addition, phage enzymes such as endolysins (e.g., Ply40, PlyLM, A10, and LysZ5) can effectively degrade biofilms and eliminate bacteria without affecting food quality.

The effectiveness of phage treatment depends on several factors, including the type of food, the production areas in the plant, the initial contamination levels, and the specific *Listeria* strains present in the product, as phage performance can vary among different pathogen strains. While phages alone are not a comprehensive solution for addressing *Listeria* contamination in food, their effectiveness is significantly enhanced when combined with other factors. Despite the progress made, further research is necessary to explore phage-based *Listeria* inhibition in biofilms. Additionally, studies on phage resistance mechanisms and continuous monitoring of phage-resistant strains in food processing plants where phage products are permitted are crucial. Implementing such measures will help ensure the long-term effectiveness of bacteriophages and enhance food safety.

## Data Availability

No new data were created or analyzed in this study.
